# Does going against the norm on women’s economic participation increase intimate partner violence risk? A cross-sectional, multi-national study

**DOI:** 10.1186/s41256-024-00399-2

**Published:** 2024-12-26

**Authors:** Anaise Williams, Lori Heise, Nancy Perrin, Colleen Stuart, Michele R. Decker

**Affiliations:** 1 Department of Population, Family and Reproductive Health, Johns Hopkins Bloomberg School of Public Health, Baltimore, USA; 2 Johns Hopkins Carey Business School, Baltimore, USA; 3 Prevention Collaborative, Antigua, Guatemala; 4 Johns Hopkins School of Nursing, Baltimore, USA

**Keywords:** Intimate partner violence, Women’s economic empowerment, Gender norms

## Abstract

**Background:**

Women’s economic empowerment (WEE) is believed to reduce the risk of intimate partner violence (IPV), yet the relationship between WEE and IPV has proven to be highly variable. Little attention has been given to how the normative WEE environment may influence this relationship across different settings. This study tests whether IPV is associated with Vanguard WEE, defined as individual economic participation that deviates from community norms.

**Methods:**

This cross-sectional study draws on Demographic and Health Surveys conducted in 44 low- and middle-income countries. The analytic sample was partnered women who participated in the domestic violence module, living in communities with sufficient data to construct WEE norms (n = 186,968). The relationship between Vanguard WEE—measured by the number of WEE activities a woman engaged in that were non-normative in her community—and the incidence of past-year physical IPV, sexual IPV, and partner control was evaluated using a mixed-effects multilevel logistic model. The study also explored interactions between Vanguard WEE and household wealth.

**Results:**

Women who did not deviate from the community norm had an adjusted probability of 0.15 for experiencing physical IPV in the past year. However, this probability increased to 0.17 (marginal effect (ME): 0.014; 95% CI 0.007,0.021), 0.17 (ME: 0.020; 95% CI 0.010,0.030), and 0.19 (ME: 0.037; 95% CI 0.022,0.051) for women with one, two, and three or more vanguard WEE items, respectively. Physical IPV associated with vanguard WEE was higher among poorer women (p = 0.021). Additionally, the probability of past-year sexual IPV and current partner control increased from 0.05 to 0.08 (p < 0.001) and from 0.38 to 0.44 (p < 0.001), respectively, for women with three or more vanguard WEE items.

**Conclusions:**

The study provides evidence of partner backlash in the form of IPV among vanguard women—those whose economic activities contradicted local norms. Programs designed to economically empower women in contexts where such participation is non-normative should include mechanisms to monitor and mitigate potential backlash.

**Supplementary Information:**

The online version contains supplementary material available at 10.1186/s41256-024-00399-2.

## Background

Intimate partner violence (IPV) is a persistent global health challenge, encompassing physical, sexual, and emotional violence by a sexual or romantic partner [1]. A 2022 systematic review estimated that approximately 27% of ever-partnered women worldwide experience physical and/or sexual IPV in their lifetime, with 13% experiencing it in the past year [2]. Beyond injury and death, IPV is associated with a range of adverse physical and mental health outcomes, including depression, suicide, and HIV/AIDS [3]. IPV is widely recognized as a probabilistic event resulting from the interaction of factors across the socio-ecological model [4]. From a feminist perspective, violence against women is viewed as an expression of patriarchal dominance, resulting from widespread gender inequality [5].

Women’s economic participation affects IPV risk [6], and three primary theories explain how women’s economic participation may decrease IPV. The first is related to household stress, where an increase in household income and financial stability reduces conflict within a couple [7]. The second draws on Marital Dependency Theory, suggesting that financially independent women are more likely to leave a harmful relationship [8]. The third relates to capability impact, proposing that economically empowered women are more likely to have strong social networks, enhanced self-efficacy, and improved bargaining power, enabling them to better navigate relationships, seek help, and formulate exit strategies [9]. As these channels contribute to women’s safety and gender equality more broadly, efforts to promote women’s economic empowerment (WEE) have increased substantially in recent years [10]. WEE is broadly defined as the process by which women gain access to and control over financial assets and income-generating opportunities, thereby achieving economic participation and agency comparable to that of men [11, 12].

However, theory also suggests that WEE can increase IPV due to male backlash. This may occur when a partner uses violence to reassert authority within the relationship, perceiving his status as an economic provider to be threatened or as a reaction to the stigma of having an economically active wife within the community [13, 14]. Empirical evidence supports this, with studies showing instances of increased IPV following WEE interventions. For example, a recent review of randomized controlled trials evaluating WEE interventions found cases of significantly increased IPV, particularly around partner controlling behavior [15]. A review of thirteen studies examining the correlation between microfinance membership and IPV found varied results, with some studies showing negative correlations, others finding no correlation, and some reporting positive correlations [16]. Another review of asset ownership found negative associations with IPV in three countries and positive associations in five countries [17]. Most of these studies, however, did not actively measure or control for community norms. Reflecting the dearth of analysis on the impact of WEE norms, few studies have explored potential community-level mechanisms that could explain spousal backlash against women’s economic participation.

Gaps in knowledge also exist on where, when, and to whom male backlash against WEE occurs [15]. The existing evidence has largely focused on the individual level, with less attention given to the role of local context on women’s economic participation [18]. Important work by Schuler and colleagues in Bangladesh suggests that male backlash is context-dependent, specifically, backlash can occur when a woman’s behavior is transgressive [19]. Further, Heise and Kotsadam [20] examined the distribution of survey-level prevalence of women working across Demographic and Health Surveys (DHS) surveys, finding that a woman’s risk of IPV due to paid work was greater in countries where women’s overall labor force participation was lower. Research suggests that the prevalence of IPV in response to WEE increases following an inversed U-shaped curve, with initial male backlash to changing norms eventually giving way to male acceptance and appreciation of women’s economic contributions [21]. Together, these findings suggest that women’s risk of IPV is associated with the extent that her economic participation is normative, and highlight the need for more systematic measurement of WEE norms across settings. The objectives of this study are to (1) test the hypothesis that IPV risk is higher among economically empowered women who do not conform to local norms for women’s economic participation, and (2) assess whether household wealth moderates this relationship. The findings aim to offer justification and guidance for avoiding unintentional harm from global WEE programming.

## Methods

### Study design

To better understand the heterogenous relationship between WEE and IPV, this cross-sectional study examined whether transgressing gendered norms around economic power, participation, and providership increased the risk of IPV among currently partnered women across 44 low- and middle-income countries. We used a recently developed “Vanguard WEE Index” which captures the extent to which a woman deviates from the WEE norm within her geographic community [22]. We also explored household wealth as a potential moderator, hypothesizing that higher household wealth might mitigate the relationship between a woman's vanguard status and IPV by reducing the salience of WEE norms and, consequently, the likelihood of partner backlash.

### Data source and study population

This study used data from the DHS conducted in 44 low- and middle-income countries [23]. Inclusion criteria for country surveys were: (1) conducted since 2013 for time relevance; (2) inclusion of the domestic violence module; and (3) inclusion of all eight WEE variables. The latest survey was used if a country had multiple surveys since 2013. The DHS sample is representative at the national, urban/rural residence and regional levels [23]. The DHS stratification method divides the sampling frame into sampling strata, typically based on country-defined region and urban/rural status, aiming to reduce sampling errors. The household survey employs a two-stage cluster sampling procedure where primary-sampling units (PSUs) are selected within each stratum based on probability proportional to size. Across countries, DHS sampling uses a similar strategy for identifying PSUs, in which recent census data are used to create geographic demarcations, usually falling within urban/rural strata [23]. A complete household listing is acquired for each PSU, and 20–30 households are selected by equal probability systematic sampling. All women aged 15–49 within selected households complete the women’s questionnaire. The domestic violence module is conducted with only one woman per household. As in other studies using DHS data, we approximated the descriptive norm using the geographic community as defined by the PSUs and assuming that the prevalence of the behavior within the community reflects the descriptive norm [24–27].

The sample for constructing national and sub-national prevalence for WEE measures included individual women with at least one non-missing WEE item, regardless of whether they participated in the domestic violence module (n = 1,396,783). The sample for constructing community WEE prevalence was restricted to communities where at least nine women were surveyed for each WEE measure. Although there is no established guideline for this cutoff, nine was used to maintain sufficient sample sizes per country and is comparable with past studies that report minimum cutoffs ranging from 5 to 20 [28]. The analytic sample was restricted to currently partnered women with all non-missing WEE and covariate measures, who live in communities with at least nine women surveyed for each WEE item, and who completed the domestic violence module (n = 186,968, 44 countries). Samples varied by outcome due to missing values within the domestic violence module: physical IPV (n = 184,621, 42 countries), sexual IPV (n = 170,555, 40 countries), and partner control (n = 184,725, 44 countries).

### Variables and measurement

#### Outcome measures

Past-year physical and sexual IPV measures from the DHS multi-country study, adapted from the Conflict Tactics Scale, were applied as binary measures [29]. The measure of past-year physical IPV was indicated if the participant reported that her partner had pushed or shaken, slapped, punched, kicked or dragged, strangled or burnt her, or twisted her arm or pulled her hair in the past year. The measure of past-year sexual IPV was indicated if the participant was forced to have sex or engage in unwanted sexual acts by her partner in the past year. The measure of current partner control was indicated if the respondent reported that her partner did not, at the time of survey, permit the respondent to meet with female friends, limited the respondent’s contact with family, or insisted on knowing where the participant was at all times.

#### WEE measure

The WEE variable included eight proxies of economic empowerment commonly used in the literature: (1) worked in the past year vs. did not, (2) earned the same or more than their husband vs. earned less than their husband or had no income, (3) had above primary education vs. did not, (4) worked in a professional/technical/managerial position vs. did not hold this type of position or did not work, (5) decided alone about how to spend her earnings vs. did not or had no earnings, (6) participated in decisions on how to spend husband’s earnings vs. did not or husband had no earnings, (7) participated in household purchase decisions vs. did not, (8) decided alone about seeking her own healthcare vs. did not. The WEE measure counts the number of WEE items (0–8) a woman reported having at the time of the survey (*M* = 2.68, *SD* = 1.68).

#### Vanguard WEE Index

The Vanguard WEE Index is an individual-level count measure of the number of WEE items (out of 8) for which a woman is vanguard on (*M* = 0.99, *SD* = 1.15). Being vanguard on an item was operationalized as having an item in a community where it is non-normative for women to have that item. Meaning, being vanguard on an item was a binary measure of having the item and living in a non-normative community compared to either: (1) having the item and living in a community that is normative on that item; or (2) not having the item. To identify whether a woman’s WEE item is vanguard, we applied a strategy for labeling a community as non-normative or normative for that WEE item based on the community’s prevalence for that WEE item. A community was labeled non-normative for a WEE item if either (1) the community item prevalence was < 35% or (2) item prevalence was ≥ 35% and ≤ 65% and the community item prevalence was in the bottom two-thirds of the community-level distribution within the geographic administrative area one level below national, drawing on similar methods used in other studies [24, 30]. More details on the Vanguard WEE Index can be found elsewhere [22]. Since the count index was right-skewed, we converted the index into a categorical measure of 0, 1, 2, and 3 or more vanguard items for statistical modeling.

#### Alternative vanguard WEE indices

For sensitivity analysis, two alternative measures of the Vanguard WEE Index were used. The first used the same 35%/65% threshold for non-normative and normative communities but a different approach for assigning communities with prevalence between 35% and 65%: non-normative for an item if the community prevalence was less than the regional prevalence (*M* = 0.85, *SD* = 1.08) [24]. The second measure used the same approach for assigning middle-prevalence communities but a different threshold dyad of 25%/75% (*M* = 1.14, *SD* = 1.21). The indices were converted into categorical measures of 0, 1, 2, and 3 or more vanguard items for statistical modeling.

#### Covariates

Models adjusted for age (categorical at 5-year intervals), age at marriage (categorical at 5-year intervals), parity (categorical with 0 = no children, 1 = one to two children, 2 = two to five children, and 3 = more than five children) and rurality (0 = rural, 1 = urban). Wealth is measured using a DHS-developed index that compares households within the same country (1 = poorest, 2 = poorer, 3 = middle, 4 = richer, and 5 = richest) using data on household assets, household construction materials, and water and sanitation factors. Models also control for country economic development by including GDP at the time of DHS survey, sourced from the World Bank [31].

### Statistical analysis

All analyses used the DHS domestic violence survey sampling weights [32], re-normalized due to the different survey sizes across countries so that each country survey is equally weighted. Given the clustering, all statistical models applied random intercepts for both strata and community as well as country fixed effects. The relationship between the Vanguard WEE Index and outcomes is anticipated to vary across communities; thus, models also have random slopes for the index at the community level. Unstructured covariance was specified as not to assume that the two random-effects terms are independent.

The sample breakdown and bivariate associations were explored using the design-based F-statistic. Next, each outcome was regressed on the Vanguard WEE Index separately using a three-level mixed effects multilevel logistic regression model including country fixed effects, total WEE items, household wealth index, control variables, normalized survey weights, and random effects for strata and community. Sensitivity analyses were performed with two alternative measures of the Vanguard WEE Index. As further sensitivity analysis, the association of the Vanguard WEE Index and each IPV outcome was assessed when adjusting for the other two forms of IPV. As a robustness check, the primary model for physical IPV was ran stratifying on geographic global region and low versus middle-income countries. Interactions between the Vanguard WEE Index and household wealth were tested for each outcome and significant interactions were displayed graphically in a margins plot. All analyses were conducted using Stata 17.0 with statistical analysis set a priori at p < 0.05. All inferential estimates were presented as marginal effects.

## Results

Of the sample of partnered women, most were ages 19–35 (62.7%) and married between ages 16 and 25 (72.5%) (Table 1). About 92% had children and 58% lived in rural areas. Close to 10% of the women had no WEE items, and 58.2% had three or more of the 8 WEE items. About 41% had no vanguard items, and 13.0% had three or more vanguard items. Across the full sample, the weighted estimate of past-year physical IPV was 17.0%, past-year sexual IPV was 6.4%, and current partner control was 40.3%. In bi-variate analysis, physical IPV was negatively associated with WEE (p < 0.001) and the Vanguard WEE Index (p = 0.028). Sexual IPV was negatively associated with WEE (p < 0.001) and not associated with the Vanguard WEE Index (p = 0.154). Partner control was negatively associated with WEE (p < 0.001) and positively associated with the Vanguard WEE Index (p < 0.001) (Table 1).

**Table 1:** Sample characteristics and bivariate associations with past-year physical IPV, past-year sexual IPV, and current partner control, weighted

	Sample	Past-year Physical IPV*		Past-year Sexual IPV**		Current Partner Control***	
	Col (%)	Row (%)	p-value^+^	Row (%)	p-value^+^	Row (%)	p-value^+^
Overall	–	17.0	–	6.4	–	40.3	–
Age group			< 0.001		< 0.001		< 0.001
≤ 18 years	3.4	18.3		8.5		44.7	
19–25 years	23.1	19.1		7.3		44.8	
26–35 years	39.6	17.8		6.6		41.0	
36–45 years	26.8	15.1		5.4		36.9	
> 45 years	7.1	12.5		4.8		33.3	
Age of first marriage/ cohabitation			< 0.001		< 0.001		< 0.001
11–15 years	18.3	19.4		7.4		43.1	
16–20 years	49.5	17.8		6.9		41.5	
21–25 years	23.0	14.5		5.1		37.6	
> 26 years	9.2	14.1		4.5		35.5	
Parity			< 0.001		< 0.001		0.056
No children	7.8	13.5		4.8		38.8	
1–2 children	37.1	16.3		5.9		40.4	
2–5 children	38.4	18.0		6.8		40.9	
> 5 children	16.8	18.1		7.4		39.4	
Wealth			< 0.001		< 0.001		0.052
Poorest	17.7	19.5		7.2		39.4	
Poorer	19.0	18.7		7.4		40.7	
Middle	20.3	17.4		6.4		39.7	
Richer	21.3	16.9		6.4		41.7	
Richest	21.8	13.5		4.9		40.1	
Rurality			< 0.001		< 0.001		< 0.001
Urban	41.6	15.5		5.5		41.8	
Rural	58.4	18.1		7.0		39.3	
WEE			< 0.001		< 0.001		< 0.001
0	9.9	20.3		7.3		40.4	
1	11.5	21.9		7.7		46.0	
2	20.4	17.6		7.0		40.1	
≥ 3	58.2	15.2		5.8		39.3	
Vanguard WEE^++^			0.028		0.154		< 0.001
0	40.8	17.4		6.2		39.2	
1	27.9	17.5		6.8		40.4	
2	18.4	16.1		6.2		41.2	
≥ 3	13.0	16.2		6.5		42.4	
Observations	186,968	184,621		170,555		184,725	

^+^P-value of design-based F statistic between variable and physical IPV, sexual IPV, and partner control, adjusting for standardized survey weights and sampling design

^++^Number of items vanguard; vanguard on an item if has the item and lives in a community where the item prevalence is < 35% or lives in a community with prevalence ≥ 35% and ≤ 65% and community’s prevalence is in the bottom two thirds of the community-level distribution within the community

^*^Pushed, slapped, punched, kicked, dragged, strangled, burnt, OR arm twisted in past year

^**^Forced sex OR forced unwanted sexual acts in past year

^***^Partner does not permit meeting with female friends OR partner limits contact with family OR partner insists on knowing where participant is

In a multivariate analysis controlling for covariates and overall level of WEE, and compared to women with no vanguard items, women with one vanguard item had a 1.4-percentage point (pp) increase in physical IPV (marginal effect (ME) 0.014; 95% CI (0.007, 0.021)), a 1.0-pp increase in sexual IPV (ME 0.010; 95% CI (0.005, 0.014)), and a 2.1-pp increase in partner control (ME 0.021; 95% CI (0.011, 0.032)) (Table 2). Women with two vanguard items had a 2.0-pp increase for physical IPV (ME 0.020; 95% CI (0.010, 0.030)), a 1.2-pp increase for sexual IPV (ME 0.012; 95% CI (0.005, 0.019)), and a 3.3-pp increase in partner control (ME 0.033; 95% CI (0.021, 0.045)). Women with three or more vanguard items had a likely 3.7-pp increase in physical IPV (ME 0.037; 95% CI (0.022, 0.051)), a 2.7-pp increase in sexual IPV (ME 0.027; 95% CI (0.017, 0.037)), and a 5.8-pp increase in the probability of partner control (ME 0.058; 95% CI (0.040, 0.076)) (Table 2). A one-unit increase in WEE overall, regardless of vanguard status, was associated with a 1.1-pp decrease in the probability of physical IPV (ME − 0.011; 95% CI (− 0.014, − 0.008)), a 0.5-pp decrease in sexual IPV (ME − 0.005; 95% CI (− 0.008, − 0.002)), and a 1.4-pp decrease in partner control (ME − 0.014; 95% CI (− 0.018, − 0.009)). Sensitivity analysis adjusting for other forms of IPV did not affect the direction and did not substantially affect the magnitude or significance level. The association between vanguard WEE and physical IPV did not change when analyzing the effect among low-income and middle-income countries separately (Annex Table 1). There were small differences stratifying on global geographic region, with the strongest associations between vanguard WEE and physical IPV observed in the Middle-east and North Africa, though overall findings were consistent across regions (Annex Table 2).

**Table 2:** Mixed effects logistic regression of vanguard WEE on physical IPV, sexual IPV, and partner control, marginal effects, weighted

	Past-year physical IPV		Past-year sexual IPV		Current partner control	
	Marginal effect (95% CI)	Marginal Prob	Marginal effect (95% CI)	Marginal Prob	Marginal effect (95% CI)	Marginal Prob
*Vanguard WEE items*^+^						
0	Ref	0.15	Ref	0.05	Ref	0.38
1	0.014*** (0.007, 0.021)	0.17	0.010*** (0.005, 0.014)	0.06	0.021*** (0.011, 0.032)	0.40
2	0.020*** (0.010, 0.030)	0.17	0.012*** (0.005, 0.019)	0.07	0.033*** (0.021, 0.045)	0.41
≥ 3	0.037*** (0.022, 0.051)	0.19	0.027*** (0.017, 0.037)	0.08	0.058*** (0.040, 0.076)	0.44
*WEE items*						
Cont	− 0.011*** (− 0.014, − 0.008)	–	− 0.005*** (− 0.008, − 0.002)	–	− 0.014*** (− 0.018, − 0.009)	–
Obvs	184,621		170,555		184,725	

^*^p < 0.05; **p < 0.01; ***p < 0.001

Logistic mixed effects models account for survey weighting, country fixed effects, strata and community random intercepts, adjust for total WEE items (shown in table), age, age of marriage, parity, wealth, rurality, country GDP, and random slopes for vanguard across communities

^+^Number of items vanguard; vanguard on an item if has the item and lives in a community where the item prevalence is < 35% or lives in a community with prevalence ≥ 35% and ≤ 65% and community’s prevalence is in the bottom two thirds of the community-level distribution within the community

Sensitivity analysis using an alternative Vanguard WEE Index with the same 35%/65% threshold but a different approach to labeling middle-prevalence communities as non-normative rendered similar results (Table 3). Sensitivity analysis using a second alternative Vanguard WEE Index with a threshold of 25%/75%, in contrast to 35%/65%, rendered similar results for partner control and had significant positive associations at three or more vanguard items of 1.5-pp (ME 0.015; 95% CI (0.004, 0.027)) and 1.3-pp (ME 0.013; 95% CI (0.004, 0.021)), for physical IPV and sexual IPV respectively (Table 3). The larger proportion of communities labeled “middle prevalence” allotted by a threshold of 25%/75% led to more communities being labeled as non-normative and therefore a higher mean number of vanguard items across women. As such, the measure of “non-normative” was more loosely applied in this sensitivity measure of vanguard WEE, leading to more conservative results.

**Table 3:** Sensitivity analysis: mixed effects logistic regression of alternate vanguard WEE measures on past-year physical IPV, sexual IPV, and current partner control, marginal effects, weighted

	Past-year physical IPV	Past-year sexual IPV	Current partner control
	Marginal effect (95% CI)	Marginal effect (95% CI)	Marginal effect (95% CI)
*Vanguard WEE, using a different approach for middle-prevalence communities*^+^			
0	Ref	Ref	Ref
1	0.014*** (0.006, 0.021)	0.010*** (0.005, 0.015)	0.021*** (0.011, 0.031)
2	0.021*** (0.011, 0.031)	0.014*** (0.007, 0.022)	0.032*** (0.019, 0.045)
≥ 3	0.036*** (0.021, 0.051)	0.029*** (0.018, 0.040)	0.056*** (0.036, 0.075)
Observations	184,621	170,555	184,725
*Vanguard WEE, using a different threshold dyad*^++^			
0	Ref	Ref	Ref
1	0.005 (− 0.002, 0.013)	0.005 (0.000, 0.010)	0.017*** (0.007, 0.027)
2	0.007 (− 0.001, 0.016)	0.006 (0.000, 0.012)	0.018* (0.004, 0.032)
≥ 3	0.015* (0.004, 0.027)	0.013** (0.004, 0.021)	0.044*** (0.027, 0.061)
Observations	184,621	170,555	184,725

*p < 0.05; **p < 0.01; ***p < 0.001

Logistic mixed effects models account for survey weighting, country fixed effects, strata and community random effects, adjust for total WEE items, age, age of marriage, parity, wealth, rurality, country GDP, and allow for random slopes for vanguard across communities

^+^Number of items vanguard; vanguard on an item if has the item and lives in a community where the item prevalence is < 35% or lives in a community with prevalence ≥ 35% and ≤ 65% and community’s prevalence less than the regional prevalence

^++^Number of items vanguard; vanguard on an item if has the item and lives in a community where the item prevalence is < 25% or lives in a community with prevalence ≥ 25% and ≤ 75% and community’s prevalence is in the bottom two-thirds of the community-level distribution within the region

**Fig. 1:**
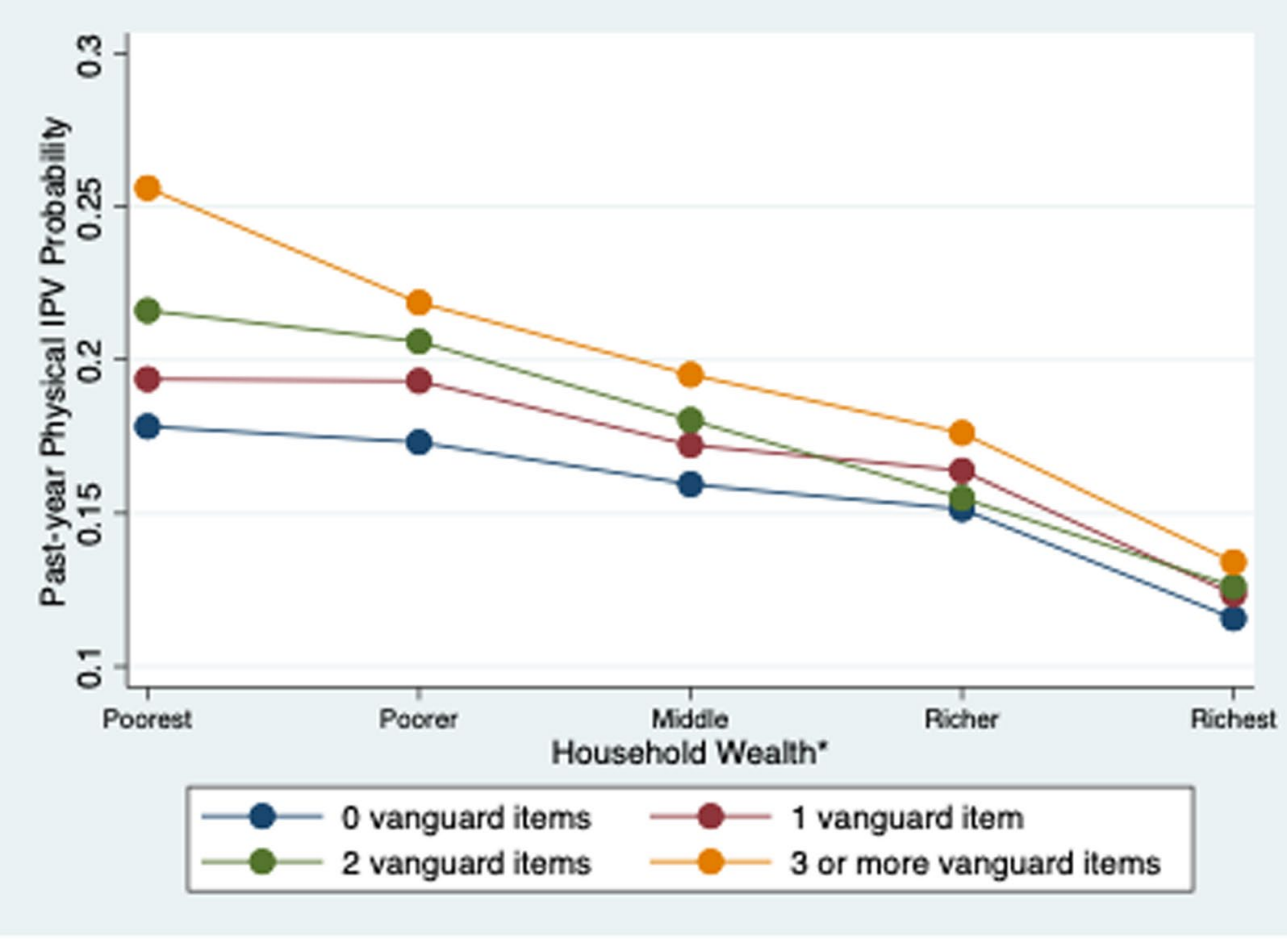
Marginal probabilities of past-year physical IPV at each wealth level, stratified by vanguard WEE (n = 184,621). *Note*: Generated by logistic mixed effects models accounting for survey weighting, country fixed effects, strata and community random effects, adjusting for total WEE items, age, age of marriage, parity, wealth, rurality, country GDP, and allowing for random slopes for vanguard across communities

Adjusting for covariates, wealth moderated the relationship between vanguard and physical IPV (p = 0.021), but not sexual IPV (p = 0.431) or partner control (p = 0.935). As shown in Fig. 1, there was a greater disparity in physical IPV by vanguard status among the poorest women. That is, wealth was associated with decreased incidence of physical IPV due to vanguard status. Among women who had the WEE items, richer women were more likely to be vanguard on employment, having an income the same or greater than husband, and agency on how to spend own income (Annex Table 3). Among women who had the WEE items, poorer women were more likely to be vanguard on higher education, professional/managerial job, and decision-making on spending husband’s income and household purchases (Annex Table 3).

## Discussion

Results suggest that “vanguard” women who go against women’s economic participation norms are at greater risk for physical and sexual IPV, as well as partner control, than peers who do not go against these economic norms. Using a new and innovative Vanguard WEE Index, our findings indicate that while overall WEE was *negatively* associated with violence, being a vanguard is *positively* associated with violence. This helps to reconcile past quantitative research that has found mixed results in the WEE-IPV relationship and demonstrates the importance of the underlying WEE norms in a community. To the best of our knowledge, our study is the first to provide critical, multi-national evidence that a reduction in IPV may occur in communities where it is more common for women to be economically active. However, when such behavior is less common, WEE can be risky for women. A second contribution of this study is the finding that vanguard behavior may be more strongly associated with past-year physical IPV among poorer women. This suggests that non-normative behavior may be less risky for women in wealthier homes. This may be partly driven by differences in vanguard status by wealth level across different WEE items, an area in need of further research. Past studies that have explored backlash against non-normative WEE typically have focused only on very low-income groups, such as those receiving microfinance programming [15, 33].

Effect sizes were relatively small, but statistically significant and noteworthy given the low prevalence, and severity, of the outcomes in consideration. A woman’s likelihood of physical IPV increased by 4-percentage points when comparing three vanguard items to none. This is an average across very large and diverse populations, suggesting it may be much higher in more specific settings. The effects of vanguard on sexual IPV were particularly high given its low prevalence. About 6% of the population reported past-year sexual IPV, and having three vanguard items compared to none was associated with a 3-percentage point increase. The strong association between partner control and the Vanguard WEE Index is also noteworthy and aligns with other work: in a recent systematic review of the association between WEE and IPV, the authors noted that male backlash often manifests as spousal controlling behavior [15]. Despite vanguard women being more empowered, these results suggest they can experience increased spousal control and monitoring, highlighting the importance of measuring psychological abuse and controlling behavior in addition to physical and sexual IPV when monitoring backlash in the context of WEE interventions.

While our largescale quantitative findings are noteworthy, we have little insight into the context in which the IPV occurred, which is a persistent gap across studies on this topic [34]. Explanations for why we observe increased risk among vanguard women is supported by qualitative work suggesting that male backlash against WEE is more likely in places where WEE is less normative. For instance, work in Bangladesh by Schuler et al. [35] found that in villages where empowerment was less normative, women who became more empowered were beaten for challenging gender norms. However, in a village with high empowerment, overall IPV was low [35]. In Uganda, a study used vignettes to assess justification of IPV and found that in scenarios where the hypothetical woman violated gendered standards of behavior, male and female participants were more likely to endorse IPV as acceptable, compared to situations where the woman did not violate standards of behavior [36]. The main theory behind this evidence is that, in many communities, being the household financial provider is a crucial component of masculinity. Such views position men who are not the primary financial provider as failures in achieving manhood, which can lead to outbursts of violence towards partners and increases in partner monitoring. This is likely heightened in places with stronger views on male economic providership, reflecting low female economic participation [37]. While the mechanisms by which vanguard WEE may lead to sexual violence are less clear and an important area for future research, it could be that men use sexual violence, like physical violence, as a way to exert control over their partners.

This study has several limitations. Endogeneity bias due to omitted variables is a persistent issue when working with cross-national datasets with limited variable overlap. There may be something different between women who seek out economic opportunity and women who do not that affects partner perpetration. Our analysis would benefit from measures on the length of time spent engaging in vanguard economic behavior, which is unavailable in this dataset. Further, vanguard women may be more likely to report IPV in a survey, compared to other women, however, recent studies have suggested that under-reporting in the DHS domestic violence module is likely minimal [38]. All analyses were cross-sectional; therefore, we cannot speak to causation, though the past-year IPV referent period, rather than lifetime IPV, helps mitigate temporality issues. The study also uses a set of WEE proxies limited by cross-national data availability. Future research could explore associations with WEE using a more culturally sensitive set of WEE proxies and include measures with more detail on quality and type of economic participation. Further, there is strength in synthesizing multiple WEE proxies into one index to capture the broader phenomenon of empowerment, but not examining specific WEE items assumes interchangeability that may not exist, and backlash may vary across economic behaviors. Future work can examine whether observed relationships are driven by specific WEE items or sub-groupings within the Vanguard WEE Index, such as agency versus resources, to further explore mechanisms and areas for intervention. There is no gold standard for measuring non-normative communities. Our measure identifies communities as normative using a proposed threshold and a previously used approach [22]. Sensitivity analysis varied the threshold and approach, though overall these findings are robust to these adjustments. Finally, the Vanguard WEE Index was constructed based on descriptive norms, or prevalence, and future research could explore the associations of interest using injunctive norms, or local attitudes towards WEE.

## Conclusions

Social change causes conflict, and this study contributes evidence that women who go against economic norms may be more likely to experience IPV. Programs aimed at economically empowering women may increase the risk of IPV in the initial stages of social change. Future WEE programming should actively measure WEE norms and set up rigorous violence monitoring systems throughout and after interventions, even if violence reduction is not a main outcome focus of the program. Monitoring systems may be most important in the early stages of interventions, or when WEE is non-normative. This study highlights the nuanced relationship between women’s economic participation and IPV across diverse settings and affirms the critical role of context.

## Supplementary Information

Additional file1 (DOCX 23 kb)

## Data Availability

The datasets generated and/or analyzed during the current study are available in the Demographic and Health Survey repository, https://dhsprogram.com/.
