# Supplementary material for: Does going against the norm on women’s economic participation increase intimate partner violence risk? A cross-sectional, multi-national study
Source: Glob Health Res Policy. 2024 Dec 26;9:53. doi: 10.1186/s41256-024-00399-2 (PMC11670509; doi:10.1186/s41256-024-00399-2)
Supplement: Supplementary file 1 — Additional file1 (DOCX 23 kb) [file 41256_2024_399_MOESM1_ESM.docx]

Annex

| **Annex Table 1: Mixed effects logistic regression of vanguard WEE on physical IPV, by country income grouping, weighted** | | |
| --- | --- | --- |
|  | Low-income Countries**^+^**  Marginal effect  (95% CI) | Middle-income countries**^++^**  Marginal effect  (95% CI) |
| **Vanguard WEE items** | | |
| 0 | Ref | Ref |
| 1 | 0.016* (0.002,0.031) | 0.011** (0.003,0.020) |
| 2 | 0.024* (0.005,0.043) | 0.016** (0.005,0.027) |
| =>3 | 0.029* (0.002,0.060) | 0.038*** (0.022,0.054) |
| Obvs | 55,497 | 129,124 |
| *p<0.05; **p<0.01; ***p<0.001  Logistic mixed effects models account for survey weighting, country fixed effects, strata and community random intercepts, adjust for total WEE items, age, age of marriage, parity, wealth, rurality, country GDP, and random slopes for vanguard across communities  ^+^World Bank Defined: Afghanistan, Burundi, Chad, DRC, Ethiopia, Gambia, Liberia, Madagascar, Malawi, Mali, Mozambique, Rwanda, Sierra Leone, Togo, and Uganda  ^++^World Bank Defined: Angola, Armenia, Benin, Cambodia, Cameroon, DR, Egypt, Guatemala, Haiti, India, Jordan, Kenya, Maldives, Mauritania, Myanmar, Namibia, Nepal, Nigeria, Pakistan, Philippines, PNG, Senegal, Tanzania, Tajikistan, Timor-Leste, Zambia, Zimbabwe | | |

| **Annex Table 2: Mixed effects logistic regression of vanguard WEE on physical IPV, by region, weighted** | | | | |
| --- | --- | --- | --- | --- |
|  | Middle East and North Africa  Marginal effect  (95% CI) | Central America  Marginal effect  (95% CI) | Sub-Saharan  Africa  Marginal effect  (95% CI) | South and Southeast Asia  Marginal effect  (95% CI) |
| **Vanguard WEE items** | | |  |  |
| 0 | Ref | Ref | Ref | Ref |
| 1 | 0.014 (-0.001,0.031) | 0.009 (-0.012,0.031) | 0.015** (0.004,0.025) | 0.005 (-0.006,0.016) |
| 2 | 0.037** (0.013,0.060) | 0.019 (-0.003,0.041) | 0.011 (-0.003,0.024) | 0.022*** (0.009,0.035) |
| =>3 | 0.055*** (0.024,0.087) | 0.035* (0.008,0.061) | 0.027** (0.07,0.046) | 0.040*** (0.020,0.060) |
| Obvs | 42,771 | 12,482 | 75,728 | 53,640 |
| *p<0.05; **p<0.01; ***p<0.001  Logistic mixed effects models account for survey weighting, country fixed effects, strata and community random intercepts, adjust for total WEE items, age, age of marriage, parity, wealth, rurality, country GDP, and random slopes for vanguard across communities  Middle East and North Africa: Afghanistan, Armenia, Egypt, Jordan, Pakistan, Tajikistan  Central America: Dominican Republic, Guatemala, Haiti  Sub-Saharan Africa: Angola, Benin, Burundi, Cameroon, Chad, DRC, Ethiopia, Gambia, Kenya, Liberia, Madagascar, Malawi, Mali, Mauritania, Mozambique, Namibia, Nigeria, PNG, Rwanda, Senegal, Sierra Leone, Tanzania, Timor-Leste, Togo, Uganda, Zambia, Zimbabwe  South and Southeast Asia: Cambodia, India, Maldives, Myanmar, Nepal, Philippines | | | | |

| **Annex Table 3: Percent vanguard by item among those who have the item, by wealth, weighted** | | | | | | |
| --- | --- | --- | --- | --- | --- | --- |
|  | Poorest | Poorer | Middle | Richer | Richest | P-value^+^ |
| **Vanguard WEE items - resources** | | |  |  |  |  |
| Employment | 31.0 | 33.3 | 36.4 | 41.5 | 44.2 | <0.001 |
| Income same or greater than husb | 75.6 | 76.8 | 80.2 | 82.4 | 89.3 | <0.001 |
| Higher education | 39.9 | 37.1 | 35.8 | 28.0 | 18.4 | <0.001 |
| Professional/managerial job | 97.9 | 97.2 | 96.2 | 92.1 | 90.8 | 0.007 |
| **Vanguard WEE items – decision making agency** | | | | | | |
| Own income | 60.9 | 59.2 | 61.8 | 66.5 | 70.3 | <0.001 |
| Healthcare access | 62.7 | 58.6 | 61.1 | 62.0 | 60.3 | 0.470 |
| Husband’s income | 32.4 | 28.3 | 27.9 | 27.0 | 22.5 | <0.001 |
| Household purchases | 27.3 | 26.1 | 22.9 | 21.1 | 17.1 | <0.001 |
| ^+^Pearson’s Design Based F statistic  Among women who have the item | | | | | | |
